# Tuberous Sclerosis Complex-1 Deficiency Attenuates Diet-Induced Hepatic Lipid Accumulation

**DOI:** 10.1371/journal.pone.0018075

**Published:** 2011-03-29

**Authors:** Heidi L. Kenerson, Matthew M. Yeh, Raymond S. Yeung

**Affiliations:** 1 Department of Surgery, University of Washington, Seattle, Washington, United States of America; 2 Department of Pathology, University of Washington, Seattle, Washington, United States of America; University of Hong Kong, Hong Kong

## Abstract

Non-alcoholic fatty liver disease (NAFLD) is causally linked to type 2 diabetes, insulin resistance and dyslipidemia. In a normal liver, insulin suppresses gluconeogenesis and promotes lipogenesis. In type 2 diabetes, the liver exhibits selective insulin resistance by failing to inhibit hepatic glucose production while maintaining triglyceride synthesis. Evidence suggests that the insulin pathway bifurcates downstream of Akt to regulate these two processes. Specifically, mTORC1 has been implicated in lipogenesis, but its role on hepatic steatosis has not been examined. Here, we generated mice with hepatocyte-specific deletion of *Tsc1* to study the effects of constitutive mTORC1 activation in the liver. These mice developed normally but displayed mild hepatomegaly and insulin resistance without obesity. Unexpectedly, the *Tsc1*-null livers showed minimal signs of steatosis even under high-fat diet condition. This ‘resistant’ phenotype was reversed by rapamycin and could be overcome by the expression of Myr-Akt. Moreover, rapamycin failed to reduce hepatic triglyceride levels in models of steatosis secondary to *Pten* ablation in hepatocytes or high-fat diet in wild-type mice. These observations suggest that mTORC1 is neither necessary nor sufficient for steatosis. Instead, Akt and mTORC1 have opposing effects on hepatic lipid accumulation such that mTORC1 protects against diet-induced steatosis. Specifically, mTORC1 activity induces a metabolic shift towards fat utilization and glucose production in the liver. These findings provide novel insights into the role of mTORC1 in hepatic lipid metabolism.

## Introduction

NAFLD represents a spectrum of changes in the liver that are closely associated with obesity, type II diabetes and other manifestations of the metabolic syndrome [1] [2]. The accumulation of triglycerides (TG) in the liver, known as steatosis, is the initial and requisite event in the pathogenesis of NAFLD [1]. Over time, steatosis may progress to steatohepatitis, which is becoming a major contributor to chronic liver disease including cirrhosis and primary liver cancers in the United States. Weight reduction (including bariatric surgery) and exercise are the only widely accepted treatments for patients with NAFLD. Recent study suggests that vitamin E and pioglitazone may be beneficial, but their long-term effects are not known [3].

The widely recognized association between NAFLD and insulin resistance suggests a role of the insulin signaling pathway in hepatic steatosis. As a growth factor, insulin activates PI3K through its interaction with the insulin receptor and its substrate, IRS1/IRS2 [4]. The catalytic function of PI3K generates second messengers (e.g., PIP_3_) to promote PDK1- and mTORC2-dependent phosphorylation of Akt, while PTEN inhibits this process by reducing PIP_3_ through its phosphatase activity [5] [6]. Once activated, Akt phosphorylates FoxO1 and inhibits the transcription of genes required for gluconeogenesis (e.g., phosphoenolpyruvate carboxykinase [PEPCK]). Insulin also stimulates lipid synthesis in the liver via SREBP1c-mediated transcription of lipogenic genes. In type 2 diabetes, hepatic glucose production becomes insensitive to insulin while TG production remains responsive resulting in selective hepatic insulin resistance [7]. Consequently, this leads to the classic triad of hyperinsulinemia, hyperglycemia and hypertriglyceridemia found in the metabolic syndrome.

Recent studies highlight the role of mTORC1 in lipogenesis and provide an understanding for the selective hepatic insulin resistance in type 2 diabetes [8]. Firstly, it was shown that Akt-dependent lipogenesis is mediated by mTORC1 through its effects on SREBP-1c [9]. This is supported by the work of Duvel et al. showing increased lipogenesis in *Tsc2*−/− cells, and the work of others highlighting the effects of rapamycin on the inhibition of multiple lipogenic enzymes (e.g., FASN, ACC, SCD-1) [10] [11] [12]. Secondly, mTORC1 participates in adipogenesis by promoting the translation of C/EBP-α, and PPAR-γ, which further direct the transcription of lipogenic genes [13]. Thirdly, SREBP1c, but not PEPCK, expression was stimulated by mTORC1 in the liver suggesting that mTORC1 may be the bifurcation point for insulin action on lipid and carbohydrate metabolism [14]. Fourthly, published reports show that events up-stream (i.e., loss of PTEN [15], [16] and LKB1 [17], activation of Akt [18]) and down-stream (i.e., loss of 4E-BP1/2 [19], activation of SREBP1 [20]) of mTORC1 can induce steatosis. Finally, animal models of obesity and type II diabetes such as the Zucker rat and the ob/ob mouse, as well as diet-induced models of steatosis all share in the dysregulation of the Akt/mTOR pathway [21] [22] [23]. Collectively, the current view suggests that mTORC1 promotes SREBP1-dependent lipogenesis while it suppresses Akt-mediated effects on gluconeogenesis; however, direct evidence causally linking mTORC1 to the pathogenesis of steatosis remains lacking.

mTOR (mammalian target of rapamycin) is a highly conserved serine/threonine kinase that plays a central role in the regulation of cell growth in response to environmental cues: energy, nutrients, stress, and oxygen [24]. It forms two known multi-protein complexes: mTORC1 consisting of mTOR, mLST8, Raptor, Deptor and PRAS40, and mTORC2 containing mTOR, mLST8, mSin1, Rictor, Deptor and Protor-1. Upon activation by Rheb-GTPase, mTORC1 enhances protein translation and ribosomal biogenesis in a rapamycin-sensitive manner. In contrast, mTORC2 phosphorylates Akt, SGK1, and PKC, and is insensitive to rapamycin. Studies have identified a central role of the tuberous sclerosis complex (TSC1/TSC2) in negatively regulating mTORC1 function by serving as a GTPase activating protein to suppress the activity of Rheb [25] [26] [27] [28]. Phosphorylation of TSC2 by Akt, ERK and RSK in response to growth factors results in mTORC1 activation [29]. TSC1 functions to stabilize TSC2 and maintains its proper subcellular localization [30] [31]. Hence, the loss of either TSC1 or TSC2 gives rise to persistent mTORC1 activity, which initiates a negative feedback via S6K1 phosphorylation of IRS to suppress PI3K signaling [32]. In the absence of TSC1 or TSC2, cells possess high mTORC1 and low Akt activities; this provides a unique opportunity to examine the function of mTORC1 without the concomitant influence of Akt.

In this study, we examined the *in vivo* consequence of mTORC1 activation in the liver by genetically ablating *Tsc1* in hepatocytes. To our surprise, liver-specific *Tsc1*−/− mice not only failed to show evidence of steatosis, but also were resistant to triglyceride accumulation in the liver when challenged with a high-fat diet. We further demonstrate that mTORC1 is neither sufficient nor necessary for steatosis, and that Akt and mTORC1 activities have opposing effects on hepatic accumulation of TG. These findings provide additional insights into the role of the Akt/mTORC1 pathway in hepatic lipid metabolism.

## Methods

### Ethics Statement

All animal work was conducted in accordance with national guidelines and has been approved by the Institutional Animal Care and Use Committee (IACUC) at the University of Washington under protocol 3051-03. Euthanasia was performed using compressed carbon dioxide (CO2) from gas cylinders or cervical dislocation under anesthesia. We adhered to all policies and recommendations from the American Veterinary Medical Association.

### Chemicals and antibodies

Rapamycin was obtained from Calbiochem (now EMD, Gibbstown, NJ). Antibodies for HA tag, Tubulin, and Actin were purchased from Sigma (St. Louis, MO). Antibodies for Adipophilin and β-galactosidase were purchased from Promega (San Luis Obispo, CA). The Tsc2 antibody was purchased from Santa Cruz Biotechnology Inc. (Santa Cruz, CA) and the antibody for Total GSK-3β was purchased from BD Biosciences (San Jose, CA). All other antibodies were purchased from Cell Signaling Technology (Danvers, MA).

### Animals

*Tsc1^flox/flox^* mice were obtained from David Kwiatkowski at Brigham and Women's Hospital (Boston, MA). *Pten^flox/flox^* (#006068) and *Alb-Cre* (#003574) mice were purchased from Jackson laboratories (Bar Harbor, ME). *Tsc1^flox/flox^* and *Pten^flox/flox^* mice were bred with *Alb-Cre* mice to generate progeny with hepatocyte-specific *Tsc1* and *Pten* deletion respectively. Wild-type littermates were used as controls. All experiments were done in accordance with the IACUC at the University of Washington, Seattle.

For characterization of the *Tsc1*-null and *Pten*-null livers, mice were fasted overnight (less than 12 hours). For acute rapamycin treatment, mice received an intraperitoneal injection of rapamycin (2 mg/kg) or vehicle control six hours prior to sacrifice. For rapamycin-treated *Pten*−/− mice, male mice were treated with an intraperitoneal (IP) injection of 2 mg/kg of rapamycin (diluted in DMSO) or DMSO vehicle once daily Monday through Friday for two weeks, starting at 12 weeks of age. For *Tsc1*−/− mice challenged on high-fat diet, six-week old, female mice were placed on either the Surwit high fat diet (HFD) or normal chow diet (NCD) for six weeks. The rapamycin treated cohort of *Tsc1*−/− mice on HFD was administered 2 mg/kg of rapamycin or vehicle control (M,W,F) starting at week eight for four weeks. Body weights were monitored weekly and animals were fasted before sacrifice.

### Diet-induced Steatosis

Male, wild-type C57BL/6J mice were purchased from Jackson Laboratories (Bar Harbor, ME). At eight weeks of age mice were placed on either NCD (PicoLab Rodent Diet 20, 5053) composed of 25% protein, 13% fat, and 62% carbohydrate, from LabDiet or the Surwit HFD, (D12330, 16.4% protein, 25.5% carbohydrate, 58% fat) purchased from Research Diets Inc. (New Brunswick, NJ). Mice were divided into five groups: NCD for six weeks, HFD for six weeks, HFD for 4 weeks with reversion to NCD for two weeks, NCD for six weeks with rapamycin treatment for last two weeks, and HFD for six weeks with rapamycin treatment for the last two weeks. Mice were treated with 2 mg/kg of rapamycin (M, W, F) for two weeks.

### Adenovirus Injection

*Tsc1*−/− mice were injected with 1×10^7^ plaque-forming units per gram of body weight of either Myr-HA-Akt1 or β-gal control adenovirus (Vector Biolabs, Philadelphia, PA) in 200 µl of saline via tail vein injection. Four days after injection fasted mice were sacrificed and tissues procured for analysis.

### Glucose Tolerance Test and Insulin Sensitivity Tests

For Glucose Tolerance Test (GTT), mice were fasted for sixteen hours and weighed. After sixteen hours, fasting blood glucose was obtained from venous blood via tail nick and measured with OneTouch blood glucose monitoring system and test strips from LifeScan, Inc. (Milpitas, CA). Mice received an IP injection of glucose (1 mg/g body weight). Blood glucose values were obtained at 15, 30, 60, and 120 minutes. At 30 minutes 50 µl of blood was procured for insulin assay. For the Insulin Sensitivity Tests (IST) mice were fasted for four hours and weighed. After fasting, a blood glucose level was obtained at time 0 and then 0.5 mU/g of insulin was given via IP injection and additional blood glucose values were obtained at 15, 30, 60, and 120 minutes.

### Systemic and hepatic insulin response

*Tsc1−/−* and *Tsc1+/+* mice were fasted for eight hours before insulin injection. Thirty minutes prior to insulin injection mice were anesthetized with ketamine/xylazine. Mice were given 0.5 mU/g of insulin (Lilly, Indianapolis, Indiana) in 500 µl of saline or just 500 µl of saline alone via IP injection. Ten minutes after injection, mice were euthanized via cervical dislocation. Tissues were procured immediately upon sacrifice and processed for protein analysis.

### Western Blot

Mouse liver, white adipose tissue (WAT), and muscle were homogenized in ice-cold radioummunoprecipitation (RIPA) buffer (1% Nonidet P-40, 1% sodium deoxycholate, 0.1% SDS, 0.15 M NaCl, 10 mM Tris (pH 7.2), 0.025 M β-glycophosphate (pH 7.2), 2 mM EDTA, and 50 mM sodium fluoride) with protease and kinase inhibitors (0.05 mM AEBSF, 10 µg/ml aprotinin, 10 µg/ml pepstatin, 1 mM orthovanadate, 10 µg/ml leupeptin, 1 mM microcystin LR). The protein concentration was measured using the BCA Protein Assay (Pierce, Rockford, IL). Equal amounts of protein were separated by SDS-PAGE, transferred to Immobilin-P membranes (Millipore, Bedford, MA) and blotted with antibodies according to manufacturer recommendations.

### Histology

Slides were deparaffinized, rehydrated, and washed before staining with hematoxylin QS and eosin (Vector Laboratories, Burlingame, CA) and mounting with Permount (Fischer Scientific, Santa Clara, CA). For Oil Red O staining, 5 micron thick frozen sections were cut and stained with Oil Red O diluted in propylene glycol.

### Plasma Metabolic Parameters

Blood was extracted via cardiac puncture immediately after sacrifice. Blood was spun for 15 minutes at 3000 rpm at 4°C. Plasma was analyzed for glucose, triglycerides, leptin, adiponectin, and insulin. Plasma insulin, leptin, adiponectin were quantified using Luminex and Linco Elisa Kits (Millipore, Billerica, MA). Plasma triglycerides were quantified via a colorimetric assay using a triglyceride assay kit from Roche Diagnostics.

### Liver Triglyceride Analysis

Lipids were extracted from 100 mg of liver tissue using chloroform:methanol and solubilized in 1% triton X-100/chloroform (v/v) [33]. Liver triglycerides were quantified via a colorimetric assay using a triglyceride assay kit from Roche Diagnostics.

### Real-time PCR

Total RNA was extracted from fresh/frozen liver tissue using TRIzol (Invitrogen, Carlsbad, CA) according to the manufacture's instructions. Three micrograms of RNA was reverse transcribed using Promega M-MLV Reverse Transciptase. PCR for sterol regulatory element binding protein 1c (SREBP1c) (PrimerBank ID 14161491a1), adipose triglyceride lipase (ATGL), fatty acid synthase (FASN) (PrimerBank ID 309119099a1), glucokinase (GK) (PrimerBank ID 31982798a1), phosphoenolpyruvate carboxykinase1 (PEPCK) (PrimerBank ID 7110683a1), peroxisome proliferator activated receptor gamma (PPARg) (PrimerBank ID 6755138a1), PPAR gamma co-activator (PGC1) alpha (PrimerBank ID 6679433a1), apolipoprotein B (ApoB) (PrimerBank ID 930134a1), microsomal triglyceride transfer protein (Mttp) (PrimerBank ID 6678960a1) ribosomal protein L32 (L32 sybr) (PrimerBank ID 25742730a1) genes were performed using the primer sequences listed in Table 1.

**Table 1:** Primer sequences used in qRT-PCR.

Gene Name	Forward	Reverse
ACLY	5′-CAGCCAAGGCAATTTCAGAGC-3′	5′- CTCGACGTTTGATTAACTGGTCT-3′
ATGL	5′- TGTGGCCTCATTCCTCCTAC-3′	5′- TGCTGGATGTTGGTGGAGCT-3′
FASN	5′- GGAGGTGGTGATAGCCGGTAT-3′	5′- TGGGTAATCCATAGAGCCCAG-3′
GK	5′- TGAGCCGGATGCAGAAGGA-3′	5′- GCAACATCTTTACACTGGCCT-3′
PEPCK	5′- CTGCATAACGGTCTGGACTTC-3′	5′- CAGCAACTGCCCGTACTCC-3′
PPARg	5′- TCGCTGATGCACTGCCTATG-3′	5′- GAGAGGTCCACAGAGCTGATT-3′
SREBP1c	5′- GCAGCCACCATCTAGCCTG-3′	5′- CAGCAGTGAGTCTGCCTTGAT-3′
PGC1alpha	5′-TATGGAGTGACATAGAGTGTGCT-3′	5′-TATGGAGTGACATAGAGTGTGCT-3′
ApoB	5′-TTGGCAAACTGCATAGCATCC-3′	5′-TCAAATTGGGACTCTCCTTTAGC-3′
Mttp	5′-CTCTTGGCAGTGCTTTTTCTCT-3′	5′-GAGCTTGTATAGCCGCTCATT-3′
L32 SYBR	5′- TTAAGCGAAACTGGCGGAAAC-3′	5′- TTGTTGCTCCCATAACCGATG-3′

### Statistical analyses

Quantitative data were analyzed by unpaired t-test. A p-value of less than 0.05 was considered significant.

## Results

### A. Liver-specific ablation of *Tsc1* leads to hepatomegaly and insulin resistance

**Figure 1:**
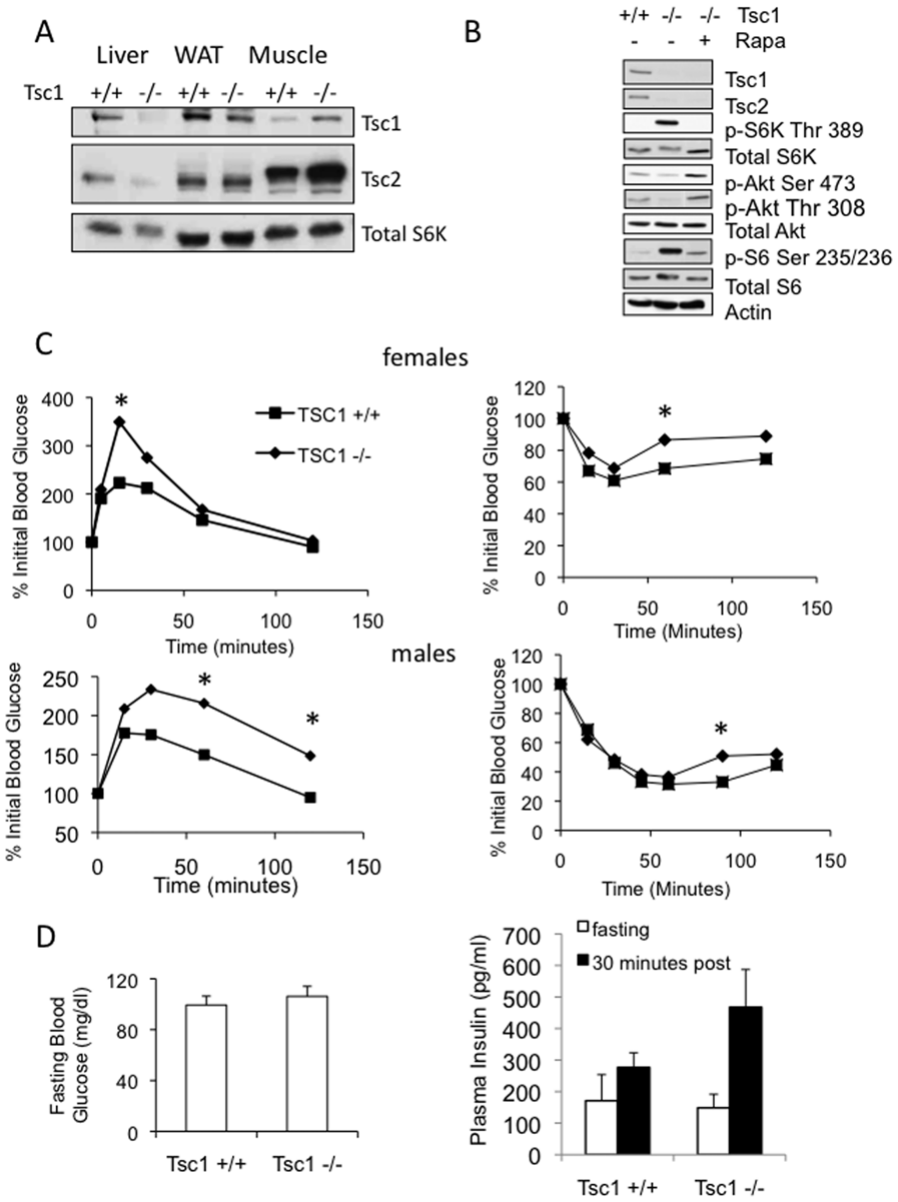
Hepatocyte-specific deletion of Tsc1 leads to mild insulin resistance. A. Liver-specific ablation of *Tsc1*. *Tsc1^fl/fl^* mice were crossed to *Cre^Alb^* mice resulting in *Tsc1^fl/fl^*; *Cre^+/+^* (a.k.a. *Tsc1*+/+) and *Tsc1^fl/fl^*; *Cre^Alb^* (a.k.a. *Tsc1*−/−) littermates. Tissues from eight-week old *Tsc1*−/− and *Tsc1*+/+ animals were analyzed for the expression of Tsc1, Tsc2 and S6K by immunoblot analyses using the indicated antibodies. WAT, white adipose tissues. Note the reduced Tsc2 expression secondary to its diminished stability in the absence of Tsc1 [30]. B. The loss of *Tsc1* in hepatocytes resulted in increased mTORC1 activity (based on the expression of phospho-S6K and phospho-S6) that was sensitive to rapamycin. *Tsc1*−/− mice were fasted and treated with or without rapamycin (2 mg/kg IP, 6 hrs). Liver lysates were analyzed by SDS-PAGE and blotted with the indicated antibodies. Note the effect of rapamycin on Akt phosphorylation in the *Tsc1*−/− liver. Actin, loading control. C. Systemic glucose tolerance (left) and insulin sensitivity (right) tests in 8-week old female (top) and male (bottom) mice. Following a 16-hr fast, glucose (1 mg/g) was given IP followed by serial blood glucose monitoring at indicated times. For insulin sensitivity test, 0.5 mU/g of insulin was injected IP after a 4-hr fast. *, p<0.05 between the *Tsc1*+/+ and *Tsc1*−/− groups. D. Fasting blood glucose and insulin levels in wild-type and mutant mice. Plasma insulin levels 30 minutes after glucose administration are also shown (filled boxes).

To investigate the biologic effects of mTORC1 in the liver, we ablated a key negative regulator of mTORC1, *Tsc1*, in hepatocytes by crossing Albumin-Cre mice with animals carrying floxed alleles of *Tsc1*. The resulting *Tsc1^flox/flox^*;*Alb-Cre* mice (a.k.a. *Tsc1*−/−) showed liver-specific loss of *Tsc1* that was accompanied by a reduction in steady-state Tsc2 expression secondary to the known influence of Tsc1 on Tsc2 stability [30] (Figure 1A). Consequently, the *Tsc1*−/− livers possessed constitutively active mTORC1 as shown by increased expression of phospho-S6K1(Thr389) and phospho-S6(Ser235/236), and this activity was inhibited by rapamycin (Figure 1B).

The *Tsc1*−/− mice developed normally and were fertile. At 20 weeks of age, average total body weight of the mutant mice was not statistically different from the wild-type mice (29.7 g vs. 27.2 g, p = 0.13), while their absolute liver weights (1.17 g vs. 0.91 g, p = 0.002) and liver:body weight ratios (3.9% vs. 3.4%, p = 0.002) were significantly higher compared to wild-type littermates consistent with hepatomegaly. The weights of the white adipose tissue (WAT) were comparable between the two groups (0.87 g vs. 0.75 g, p = 0.63). To determine systemic insulin response in these animals, glucose tolerance tests and insulin sensitivity tests were performed. Following systemic administration of glucose, both male and female *Tsc1*−/− mice exhibited mild but significant glucose intolerance compared to *Tsc1*+/+ mice (Figure 1C). This was accompanied by a slight reduction in systemic insulin sensitivity (Figure 1C). Fasting plasma glucose and insulin levels were not significantly different between the *Tsc1*+/+ and *Tsc1*−/− mice, while the insulin levels at 30 minutes following glucose administration were higher in the mutant mice (Figure 1D). These observations suggest that the hepatic-specific *Tsc1*−/− mice are insulin resistant.

**Figure 2:**
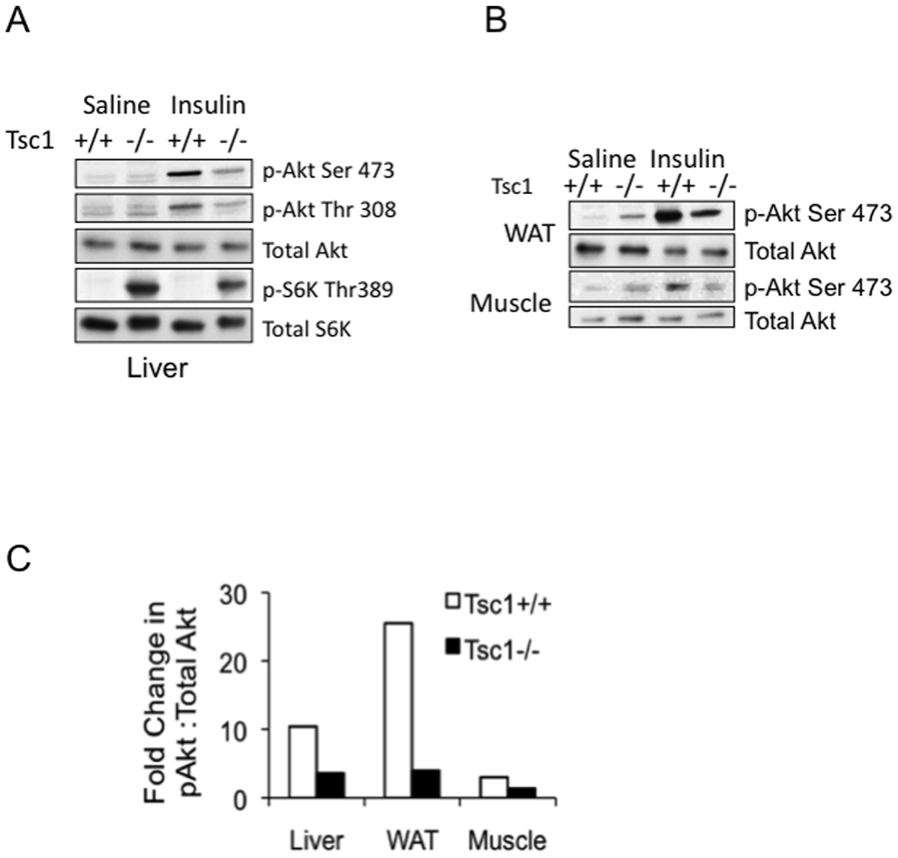
Liver, fat and muscle of Tsc1−/− mice show blunted response to insulin. Following an eight-hour fast, mice were injected with 0.5 U/kg of insulin or saline (control) for 10 minutes before sacrifice. A) Liver and B) white adipose tissue (WAT) and skeletal muscle tissue lysates were analyzed for the expression of indicated proteins. C. Akt response to insulin. The relative band intensities of p-Akt(Ser473) were normalized to total Akt from (A) and (B), and then the ratios between the insulin- and saline-treated animals were calculated from individual tissues tested.

**Figure 3:**
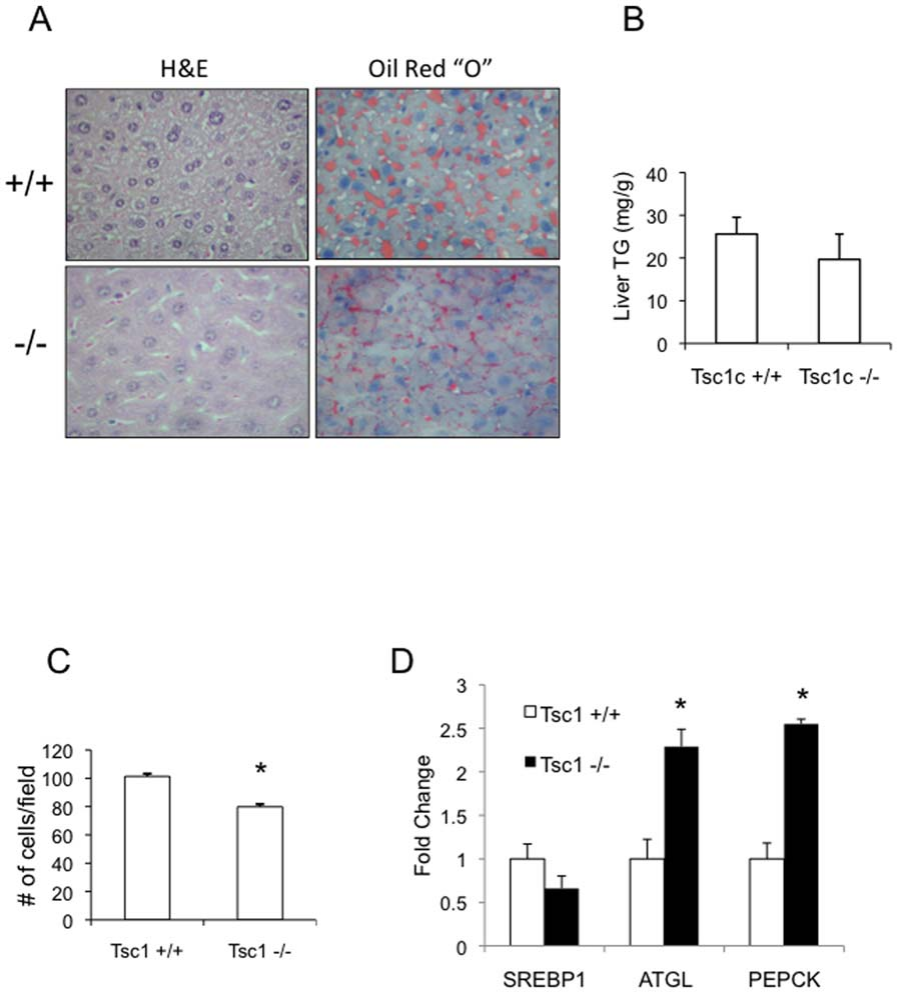
mTORC1 activity is not sufficient for steatosis. Normal chow-fed, 20-week old male *Tsc1*+/+ and *Tsc1*−/− male mice were fasted overnight and sacrificed. Liver tissues were processed for histologic and biochemical analyses. A) Liver histology (H&E) and Oil Red “O” staining showing hepatic morphology and lipid content. Magnification 400X. B) Quantification of liver triglyceride content using TG assay kit (Roche Diagnostics, see Methods). C) Hepatocyte cell size was deduced based on the average number of hepatocytes per high-power field from 10 randomly selected fields. *, p<0.01 compared to *Tsc1*+/+. D) Expression of genes involved in lipogenesis (SREBP1), adipogenesis (PPARg), lipolysis (ATGL) and gluconeogenesis (PEPCK) were determined by quantitative RT-PCR. *, p<0.05 compared to *Tsc1*+/+. For all graphs, values represent mean ±SEM.

To further examine the response to insulin in the liver, WAT and muscle tissues, fasted mice were given 0.5 U/kg of insulin 10 minutes before sacrifice. As expected, insulin led to a significant increase in Akt phosphorylation in all three tissues in the wild-type animals (Figure 2A,B). However, the Akt response to insulin was dramatically blunted in the tissues derived from the *Tsc1*−/− mice. In the case of the *Tsc1*−/− hepatocytes, hyperactivity of mTORC1 is known to inhibit Akt secondary to feedback inhibition on IRS1 [32]. Consequently, both baseline and insulin-stimulated Akt activities were suppressed in the *Tsc1*−/− liver (Figure 2A). In contrast, basal Akt phosphorylation in WAT and muscle tissues were elevated in the mutant mice relative to wild-type controls while insulin-stimulated response was significantly reduced (Figure 2B). This finding cannot be explained by the ‘feedback’ mechanism but we speculate that the chronic systemic exposure to post-prandial hyper-insulinemia (or other insulin-like growth factors) in the mutant animals may be responsible. Figure 3C highlights the relative changes in Akt(Ser473) phosphorylation between the saline- and insulin-treated wild-type and mutant animals. These results indicate that the loss of hepatic *Tsc1* leads to hepatic and systemic insulin resistance.

### C. Persistent mTORC1 activity is not sufficient for steatosis

Despite an increase in liver mass and the presence of insulin resistance, the *Tsc1*−/− mice showed no histologic or biochemical evidence of excessive TG accumulation in the liver compared with wild-type littermates (Figure 3A,B). Oil Red “O” staining and direct TG measurements suggested a trend towards reduced lipids in the *Tsc1*−/− livers. Hence, the hepatomegaly in the mutant mice was not secondary to steatosis but instead, due to a significant increase in hepatocyte cell size (Figure 3C). This is consistent with the role of mTORC1 in regulating cell growth [25]. Plasma TG levels in the *Tsc1*−/− mice were significantly lower than the wild-type littermates (55 vs. 88 mg/dl, p<0.05) while adiponectin (+/+: 4,983 pg/ml; −/−: 5,870 pg/ml, p = 0.27) and leptin (+/+: 3,814 pg/ml; −/−: 3,236 pg/ml, p = 0.66) levels were similar between the two groups. At fasting, mRNA expression of SREBP-1c involved in lipogenesis was slightly lowered while genes involved in lipolysis (ATGL) and gluconeogenesis (PEPCK) were significantly elevated in the *Tsc1*−/− mice compared to wild-type animals (Figure 3D). These findings show that hyperactive mTORC1 in hepatocytes is not sufficient to induce steatosis *in vivo* despite evidence of hepatic and systemic insulin resistance.

### D. Inhibition of mTORC1 fails to reverse diet-induced steatosis

**Figure 4:**
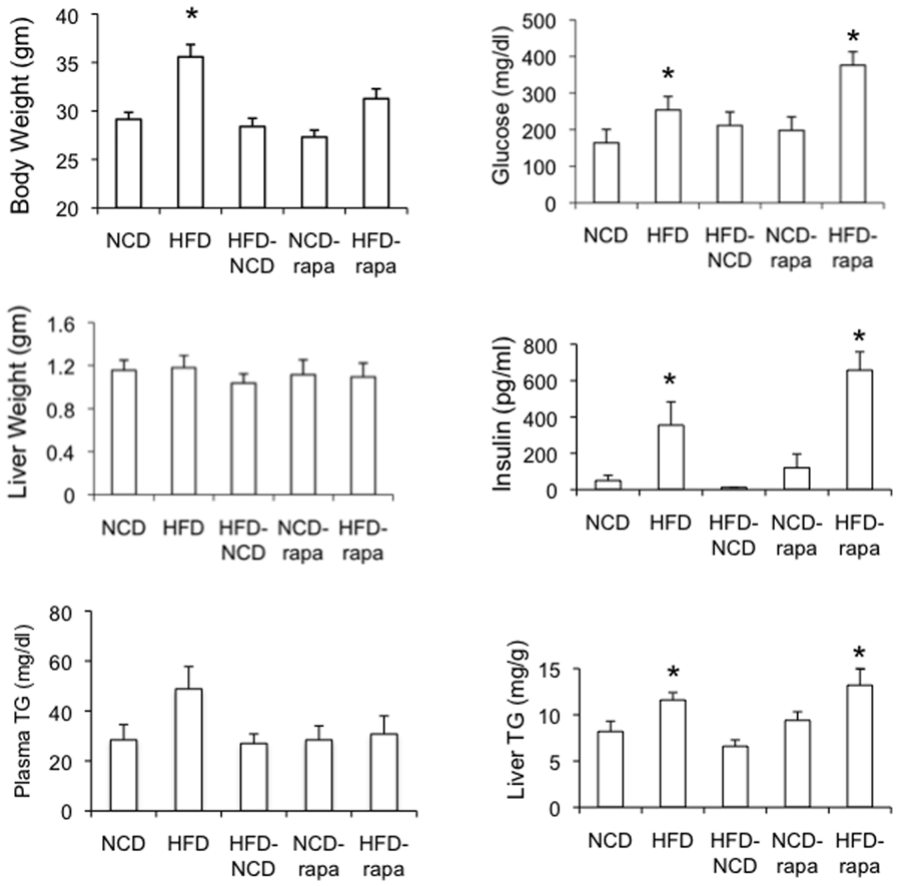
Metabolic response to rapamycin following HFD. Six-week old, wild-type mice were randomly assigned to one of 5 groups with n = 5 in each group (see text). At the end of 6 weeks, mice were fasted overnight and sacrificed. Shown are the results of body and liver weights, fasting serum glucose and insulin, plasma and hepatic triglyceride levels for each group. Values represent mean ±SEM. * associated with HFD indicates p<0.05 with respect to NCD group. * associated with HFD-rapamycin group indicates p<0.05 with respect to NCD-rapamycin group. NCD, normal chow diet; HFD, high-fat diet; Rapa, rapamycin.

Next, we set out to determine if mTORC1 activity is necessary for steatosis by examining the effects of rapamycin, an mTORC1 inhibitor, on diet-induced steatosis. Six-week old, wild-type male mice were randomly assigned to receive NCD or HFD (Surwit) for 6 weeks. Within each diet group, animals were further randomized to receive rapamycin (2 mg/kg) or DMSO (vehicle) given 3 times a week (e.g., Monday, Wednesday, Friday) via intraperitoneal injections during the last two weeks of the study. To ascertain that a two-week duration was sufficient time for a steatotic liver to recover to baseline, an additional cohort of animals was placed on HFD for 4 weeks followed by NCD for 2 weeks. Figure 4 shows that mice fed HFD sustained significantly greater weight gain compared to those on NCD as expected, but this increase was quickly lost when HFD fed mice resumed NCD. (i.e., HFD-NCD group). Rapamycin also dampened the weight gain in mice. The absolute liver weights were not significantly different among any group although the ones from the HFD-NCD-fed mice weighed the least.

HFD was associated with a significant increase in plasma levels of glucose and insulin suggestive of systemic insulin resistance, and these parameters returned to baseline within 2 weeks of resuming normal chow (Figure 4). Rapamycin treatment led to higher fasting plasma glucose and insulin levels that were most pronounced in animals receiving HFD. These findings are consistent with previous reports of glucose intolerance following chronic rapamycin treatment [34] [35].

**Figure 5:**
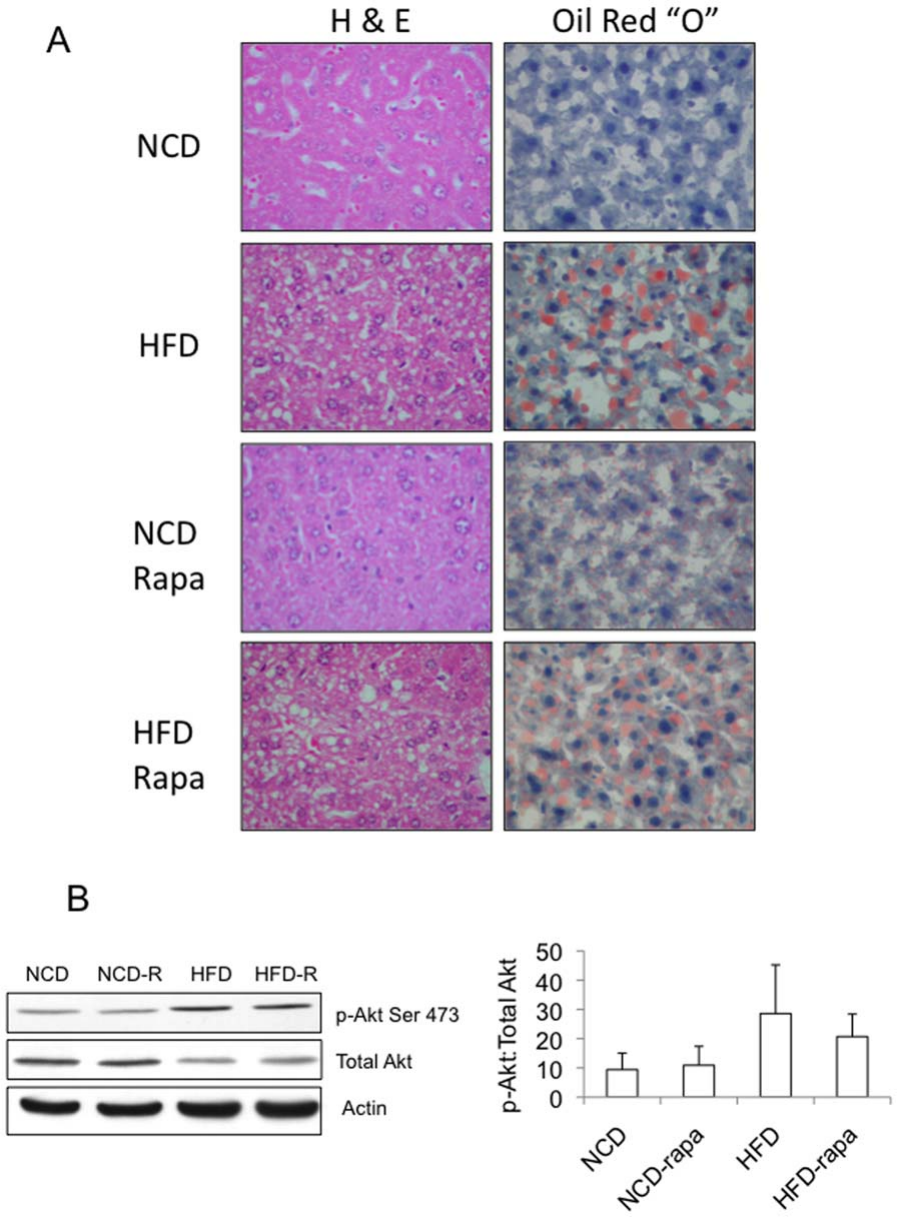
Histologic and biochemical effects of rapamycin on HFD-induced steatosis. A) Examples of histology (H&E) and Oil Red “O” staining of the livers procured from animals described in Figure 4. NCD, normal chow diet; HFD, high-fat diet; Rapa, rapamycin. Magnification, 400X. B) Western blots of representative liver lysates from each of the four groups shown in (A) highlighting the effects of chronic rapamycin on Akt(Ser473) phosphorylation. The average ratios of band intensities (Image J) between phospho- and total-Akt are summarized in the graph (n of 5 per group).

Analysis of the livers revealed a significant increase in triglyceride levels in mice receiving HFD, and importantly, rapamycin did not alter hepatic TG levels significantly in either group of animals (Figure 4). This corroborates with the histologic findings of steatosis in the HFD-groups regardless of rapamycin treatment (Figure 5A). Further, rapamycin did not lead to any histologic change in the NCD-fed livers. Immunoblot analyses of the liver lysates showed a trend towards higher levels of Akt(Ser473) phosphorylation with HFD that were unchanged following two weeks of rapamycin (Figure 5B). Hence, despite evidence of worsening glucose intolerance, mTORC1 inhibition by rapamycin did not alter hepatic lipid content in a model of diet-induced steatosis. In contrast, the HFD-associated TG accumulation and insulin resistance reverted to baseline within 2 weeks of replacing the HFD with NCD. We conclude that mTORC1 is not necessary for the maintenance of HFD-induced steatosis.

### E. Effects of rapamycin on steatosis in liver-specific *Pten*−/− mice

**Figure 6:**
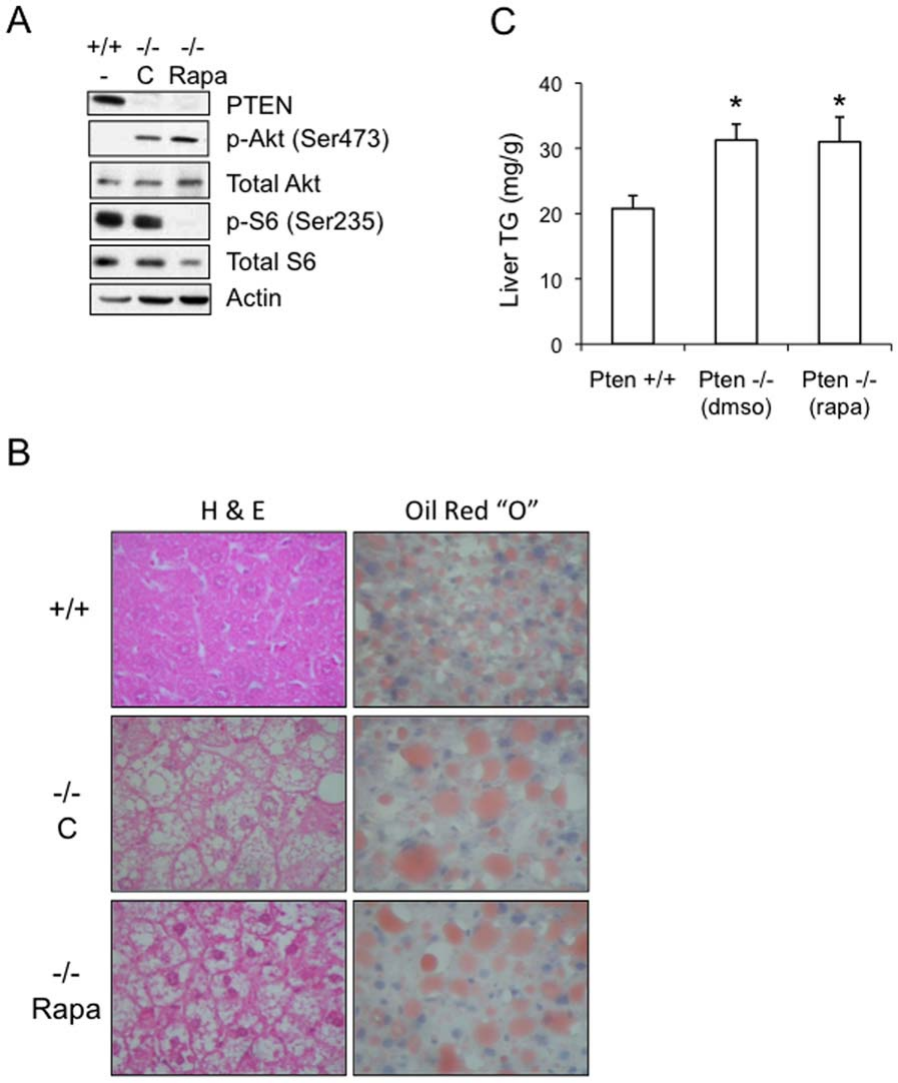
Effects of rapamycin on Pten−/− livers. Hepatocyte-specific deletion of *Pten* was generated by crossing *Pten^fl/fl^* with *Cre^Alb^* mice. At 12 weeks of age, *Pten*−/− mice were randomly assigned to treatments with rapamycin (2 mg/kg IP daily, M-F) or DMSO as vehicle control (C) for 2 weeks and then sacrificed. A) Representative Western blot showing the effects of *Pten* loss (−/−) and rapamycin (Rapa) in the liver with respect to Akt and mTORC1 signaling. Liver lysates were subjected to immunoblot analyses with the indicated antibodies. B) Liver histology (H&E) and Oil Red “O” staining of *Pten*+/+ and *Pten*−/− mice treated with rapamycin or vehicle control. C) Quantification of liver triglyceride content of the corresponding groups. Values represent mean ±SEM. *, p<0.05 compared to *Pten*+/+ group.

Given that the pathogenesis of HFD-induced steatosis may involve multiple pathways, the lack of significant change in TG accumulation following rapamycin treatment could potentially be explained by other mechanisms that affect lipid metabolism besides mTORC1. In order to focus on the relevance of mTORC1 in steatosis, we turned to an established genetic model of steatosis through *Pten* ablation in hepatocytes. We generated a cohort of *Pten^flox/flox^*;*Alb-Cre* (a.k.a. *Pten*−/−) mice that showed constitutive Akt activation in the liver (Figure 6A). At 12 weeks of age, the mutant pups had similar body weights compared to wild-type littermates but had enlarged, whitish livers containing large fat vacuoles and elevated TG levels consistent with earlier reports [15], [16]. Compared to the *Tsc1*−/− mice, the absolute liver weight and the liver:body ratio were higher in the *Pten−/−* mice while total body weight was similar in the two groups (data not shown).

Using this model, we tested whether mTORC1 activity was necessary for steatosis by treating 12-week old animals with rapamycin (2 mg/kg IP daily, M-F) or vehicle for 2 weeks. Figure 6A shows that at the end of the treatment period, rapamycin completely inhibited mTORC1 activity (as indicated by loss of phospho-S6(Ser235) expression) without significant effect on Akt activity. Histologic analysis revealed that livers of rapamycin-treated *Pten−/−* mice exhibited a similar degree of steatosis compared to vehicle-treated animals based on H&E and Oil Red “O” staining (Figure 6B). However, rapamycin reduced the average cell size of hepatocytes compared to the vehicle-treated group as quantified by the number of cells per high-power field (data not shown). This was also reflected in a reduction in liver:body weight following rapamycin treatment although it remained elevated compared to wild-type littermates (data not shown). Importantly, triglyceride content in the rapamycin-treated *Pten*−/− livers remained unchanged compared to the group treated with vehicle control, but both *Pten*−/− groups had significantly greater hepatic triglyceride concentration than the wild-type littermates (Figure 6C). Given that steatosis is rapidly reversible (e.g., see HFD-NCD in Fig. 4), these findings suggest that rapamycin affects hepatocyte cell size but not its lipid content. This led us to conclude that the inhibition of mTORC1 did not alleviate steatosis in a model where Akt is constitutively active in the hepatocytes. Further, our results indicate that Akt-induced lipogenesis is not dependant on mTORC1 activity. Together with the results of the *Tsc1*-mutant model and the effects of rapamycin in the HFD model, we deduce that mTORC1 is neither necessary nor sufficient for hepatic steatosis.

### F. The role of feedback inhibition on Akt

**Figure 7:**
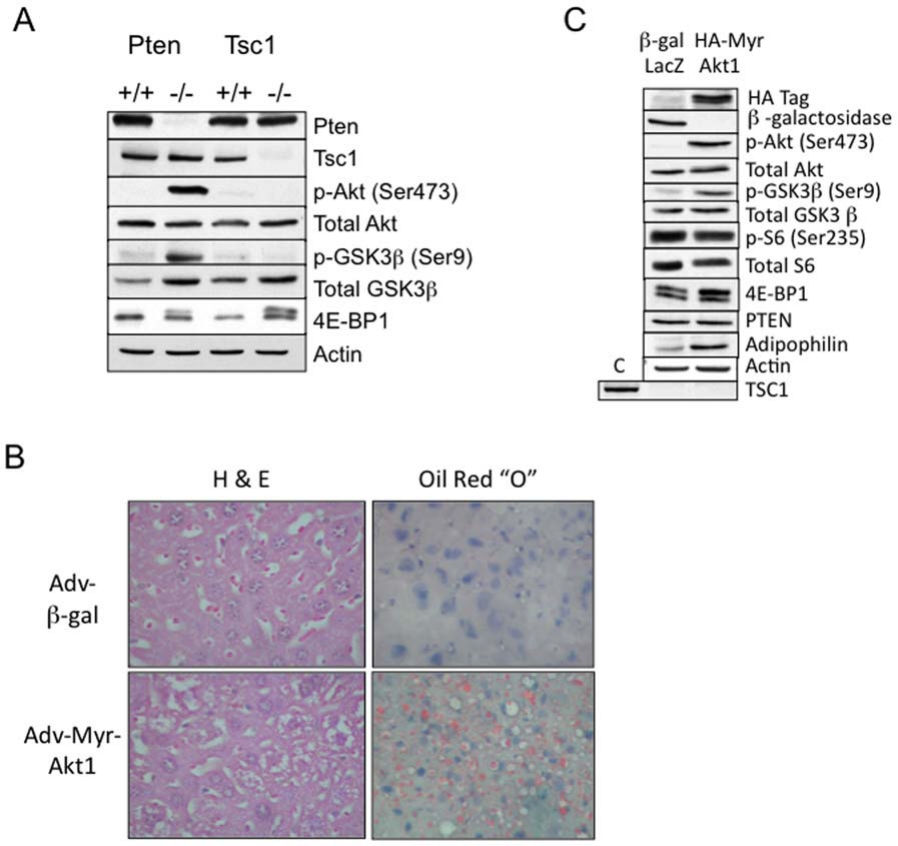
Akt induces steatosis in the Tsc1−/− livers. A) Contrasting effects of *Pten*- and *Tsc1*-loss on Akt signaling in the liver. Immunoblot analyses of liver lysates from fasted 20 wk-old mice using indicated antibodies to highlight Akt and mTORC1 signaling. B) Effects of Akt on *Tsc1*−/− livers. *Tsc1*−/− mice were injected through the tail-vein with adenovirus (10^7^ PFUs) encoding genes for Myr-Akt1 or β–galactosidase control. After 96 hours, mice were fasted overnight and sacrificed for H&E histology and Oil Red “O” staining of the livers. Magnification, 400X. C) Expression of transgenes (HA-tagged Myr-Akt1 or β–gal) and components of the Akt/mTORC1 pathway in the *Tsc1*−/− livers following adenovirus injections. C, control for Tsc1 expression. Note up-regulation of Akt without significant alteration to mTORC1 signaling in the Myr-Akt1-treated liver.

The striking differences in hepatocyte TG content of the two rodent models (i.e., *Tsc1*- and the *Pten*-hepatocyte-specific deletion) were closely paralleled by the observed disparities in their Akt activities. Figure 7A highlights the dramatic difference in Akt signaling in the livers from these two models. While Akt was markedly activated in the *Pten*-null livers, its activity was suppressed in the *Tsc1*-null state due to the negative feedback of mTORC1/S6K1 on IRS1 [32]. In both models, hepatocytic mTORC1 activity was up-regulated compared to wild-type littermates based on 4E-BP1 mobility shifts, although the *Tsc1*-null hepatocytes had significantly higher levels of mTORC1 activity compared to the *Pten*-null cells (Figure 7A). These observations led us to postulate that the relative lack of steatosis in the *Tsc1*-deficient hepatocytes may be due to the ‘feedback’ suppression of Akt. To test this hypothesis, we introduced a recombinant adenovirus encoding a constitutively active myristoylated form of Akt into the tail vein of the *Tsc1−/−* mice. Four days following injection, the Myr-Akt treated livers became significantly larger (i.e., hepatomegaly) compared to those injected with control adenovirus encoding β–galactosidase, as previously reported [18]. Microscopically, the Myr-Akt hepatocytes showed fat vacuolation consistent with steatosis and biochemically, this was associated with an up-regulation of adipophilin, a lipid droplet coat protein (Figure 7B,C). These findings were accompanied by a marked increase in Akt(Ser473) and GSK3β (Ser9) phosphorylation in the livers of the Myr-Akt treated *Tsc1−/−* animals (Figure 7C). Hence, the up-regulation of Akt in these animals was sufficient to induce steatosis. These findings also confirm that the *Tsc1*-null hepatocytes are capable of fat accumulation in an Akt-responsive manner. Together, the data suggest that the lack of steatosis in the *Tsc1−/−* mice may be secondary to the relative suppression of Akt activity.

### G. *Tsc1*−/− hepatocytes are resistant to steatosis induced by HFD

**Figure 8:**
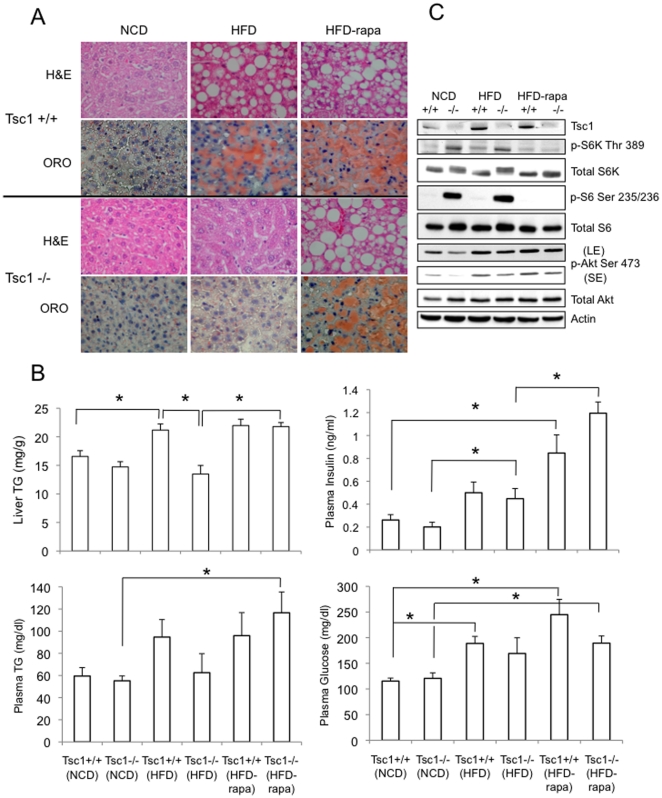
Tsc1−/− mice are resistant to diet-induced steatosis in a rapamycin-dependent manner. Six week-old *Tsc1*+/+ and *Tsc1*−/− littermates were randomly assigned to normal chow diet (NCD), high fat diet (HFD) or HFD with rapamycin (2 mg/kg IP, 3 times weekly during the last 4 weeks). At the end of 6 weeks on the assigned diets, mice were sacrifice following an overnight fast. A) H&E histology and Oil Red “O” (ORO) staining of representative liver sections from each of the 6 groups. Magnification 400X. B) Biochemical measurements of liver and plasma triglyceride (TG), plasma insulin and glucose levels are shown for each of the groups. Values represent mean ±SEM. * p<0.05. C) Western blot analyses of representative liver lysates from each group showing the effects on Akt/mTORC1 signaling. Blots from long exposure (LE) and short exposure (SE) are shown for p-Akt(Ser473). Actin, loading control.

The correlation between hepatic Akt activity and steatosis led us to predict that mTORC1-mediated suppression of Akt may protect the *Tsc1*−/− liver from TG accumulation. To test this hypothesis, 6-week old *Tsc1*−*/*− and *Tsc1*+/+ littermates were placed on a high-fat diet or normal chow diet for 6 weeks. Those assigned to the HFD were further randomized to receive rapamycin (2 mg/kg IP, MWF) or vehicle control (DMSO) during the last 4 weeks of the experiment. Over this period, weight gains on the HFD were marginally less in the mutant animals compared to wild-type littermates (p>0.10), and the liver:body weight ratio remained higher in the *Tsc1*−*/*− mice as it was under the normal diet condition (data not shown). In response to HFD, fasting plasma glucose and insulin levels rose, and rapamycin treatment further increased the values (Figure 8B). These trends suggest that glucose intolerance worsened with HFD + rapamycin in both *Tsc1*+/+ and *Tsc1*−/− mice without significant differences between the two genotypes. Plasma TG increased with HFD in the wild-type, but not mutant animals, whereas rapamycin significantly elevated plasma TG levels in the mutant but not wild-type mice (Figure 8B). On histologic analysis, the livers from the wild-type and mutant mice in the NCD group were indistinguishable, but under HFD condition, the *Tsc1*+/+ mice developed significant steatosis while the *Tsc1*−/− livers showed minimal change based on H&E and Oil Red “O” staining (Figure 8A). Direct measurements of liver TG revealed significant increase in lipid accumulation following HFD in the wild-type animals but not in the *Tsc1*−/− mice (Figure 8B). We confirmed these findings using an independent cohort of animals under the same scheme. After 6 weeks, the results of the hepatic TG levels were nearly identical with the exception that the *Tsc1*−/− mice fed HFD had significantly lower hepatic TG levels than those fed NCD (6.5 vs. 8.3 mg/g, p<0.01). Together, the histologic and biochemical evidence suggest that the loss of *Tsc1* protects the liver from TG accumulation.

To determine if the resistance to steatosis in the *Tsc1*−/− mice stems from mTORC1 hyperactivity, we treated a cohort of mice on HFD with rapamycin. The lipid content following rapamycin rose significantly in the mutant livers along with histologic evidence of steatosis (Figure 8A, B). Importantly, the levels of hepatic TG in the *Tsc1*−/− mice became equivalent to that of the *Tsc1*+/+ littermates. These rapamycin-induced phenotypic changes correlated closely with the de-repression of Akt activity (i.e., increased phospho-Akt(Ser473) expression) and the inhibition of mTORC1 activity (i.e., decreased phospho-S6(Ser235/236) expression) (Figure 8C). As noted earlier, HFD increased basal Akt phosphorylation in both wild-type and mutant livers although consistently less so in the *Tsc1*−/− samples. Up-regulation of Akt in the absence of significant mTORC1 activity (i.e., +/+ HFD, +/+ HFD-rapa, −/− HFD-rapa) was accompanied by steatosis, but when balanced by high mTORC1 activity (i.e., −/− HFD), no significant lipid accumulated (Figure 8C). These observations are consistent with our hypothesis that mTORC1 activity protects against TG accumulation in this dietary model, and that the development of steatosis is dependent on the balance between hepatic Akt and mTORC1 activities.

### H. Loss of *Tsc1* promotes fat utilization and glucose production

**Figure 9:**
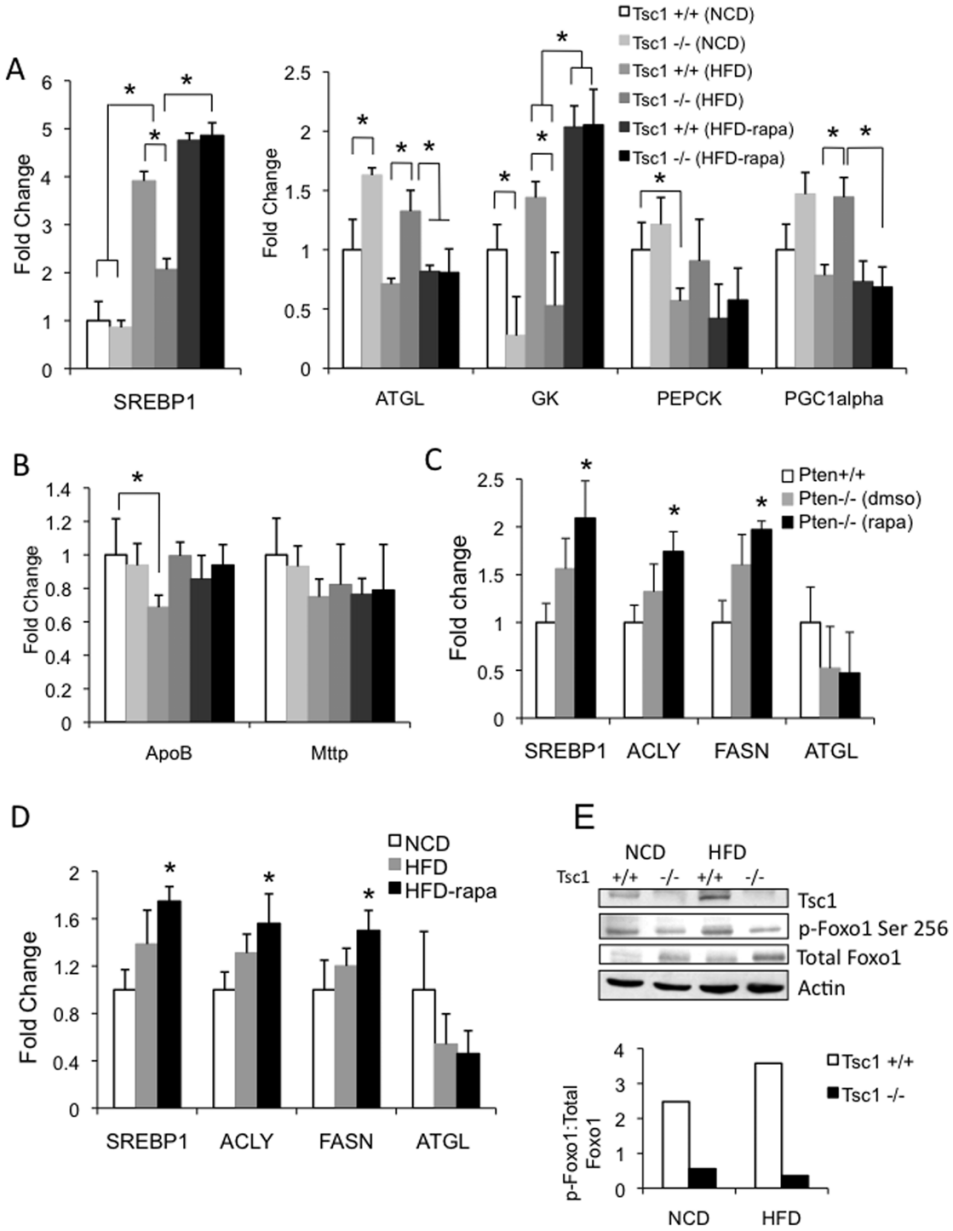
Hepatic mRNA expression of metabolic genes. Relative expression of genes involved in hepatic lipogenesis (SREBP1, ACLY, FASN), lipolysis (ATGL), gluconeogenesis (PEPCK), glycolysis (GK), mitochondrial respiration (PGC1α) and triglyceride secretion (ApoB, Mttp) were determined by RT-PCR analyses of RNA extracted from liver samples derived from experiments described in Figures 4, 6 and 8. All values represent mean ±SEM. A, B) Comparison of *Tsc1*+/+ and *Tsc1*−/− mice fed normal chow (NCD) and high-fat diet (HFD) with and without rapamycin (rapa). * p<0.05 (not all significant differences are highlighted). C) *Pten*−/− mice treated with rapamycin or vehicle (dmso) compared to wild-type littermates, * p<0.05 compared to *Pten*+/+. D) Gene expression in livers of wild-type mice fed NCD or HFD with or without rapamycin treatment. *p<0.05 compared to NCD, E) Reduced FoxO1 phosphorylation in *Tsc1*−/− livers. Tissue lysates from *Tsc1*+/+ and *Tsc1*−/− livers were analyzed for the expression of the indicated proteins by immunoblot analyses. Levels of FoxO1(Ser256) phosphorylation were quantified relative to total FoxO1 expression based on densitometric analyses (Image J).

To explore the mechanism for the steatosis-resistant phenotype in the *Tsc1*−/− liver, we analyzed the hepatic expression of genes involved in lipid and glucose metabolism by RT-PCR analyses. Figures 9A and 9B show the results from the experiments described in Figure 8 highlighting the effects of Tsc1, HFD and rapamycin on hepatic metabolic gene expression relative to NCD-fed wild-type livers. In the *Tsc1*+/+ mice, HFD induced SREBP1c and glucose kinase (GK) expression and suppressed ATGL and PEPCK expression indicative of a metabolic shift towards fat synthesis and glucose utilization leading to steatosis. The loss of *Tsc1* resulted in an opposite response to HFD with a significantly blunted increase in SREBP1c and GK expression and an exaggerated up-regulation of ATGL and PEPCK (Figure 9A). Further, these changes in the *Tsc1*−/− livers were reversed with rapamycin treatment such that the effects of mTORC1 inhibition resembled the response of the normal liver to HFD. These observations suggest that the protection from HFD-induced steatosis in the *Tsc1*−/− liver stems from a mTORC1-dependent switch in hepatic metabolism from fat synthesis to fat utilization and from glucose utilization to glucose production. Moreover, hepatic PGC1α expression was also rapamycin-sensitive and was significantly elevated in the *Tsc1*−/− livers suggestive of an increase in mitochondrial oxidation. While other factors such as TG export may influence hepatic lipid accumulation, we did not find a significant difference in the expression of hepatic microsomal triglyceride transfer protein (Mttp) between the groups although ApoB expression in the wild-type livers was significantly reduced when challenged with the HFD, a response not seen in the *Tsc1*−/− mice (Figure 9B).

In contrast, the *Pten*−/− mice have high basal SREBP1 and PPAR-γ (data not shown) expression but slightly reduced ATGL expression in the liver (Figure 9C). Treatment with rapamycin did not suppress SREBP1, ACLY or FASN expression but rather increased their levels slightly in the *Pten*-deficient livers while ATGL expression remained unchanged (Figure 9C). A remarkably similar trend in the expression of these lipogenic and lipolytic enzymes was found in HFD-induced steatosis in wild type mice suggesting that the effects of HFD closely parallel that of Akt activation (Figure 9D). Collectively, these data indicate that Akt, and not mTORC1, positively regulates a SREBP1-dependent pathway in hepatocytes *in vivo*.

The observed differences in ATGL expression in the *Tsc1*- and *Pten*-mutant livers led us to explore its up-stream regulators including PPARγ and FoxO1 that have been shown to control ATGL expression. Kim et al. reported that PPARγ directly promotes ATGL transcription, and the PPARγ agonist, rosiglitazone, leads to an increase in ATGL mRNA levels [36]. However, this mechanism is unlikely to cause the ATGL induction in the *Tsc1*−/− HFD-fed livers since high-fat diet suppressed PPARγ expression in both wild-type and mutant mice (data not shown). On the other hand, the transcription factor, FoxO1, was found to modulate lipolysis through its regulation of ATGL expression [37]. FoxO1 is an Akt target and upon phosphorylation by Akt, FoxO1 becomes sequestered in the cytoplasm as an inactive protein [38]. We examined the phosphorylation of FoxO1 at Ser256, an Akt site, using a phospho-specific antibody. Figure 9E shows that hepatic p-FoxO1(Ser256) expression was significantly reduced relative to total FoxO1 levels indicating elevated FoxO1 activity in the *Tsc1*−/− livers. These findings are consistent with the model of mTORC1-induced steatosis-resistance secondary to Akt inhibition and FoxO1 activation.

## Discussion

The notion that mTORC1 promotes lipogenesis and may contribute to NAFLD came from a series of observations showing the positive effects of mTORC1 on SREBP1 expression and activity that lead to *de novo* lipid synthesis [8] [9] [10] [11] [14] [39]. In response to insulin in the liver, Li et al. showed that mTORC1 is required for lipogenesis but is not involved in the inhibition of gluconeogenesis [14]. These and other evidence provide an understanding for the phenomenon of selective hepatic insulin resistance observed in type 2 diabetes [7]. In this study, we directly examined the effects of mTORC1 hyperactivity in genetically engineered mice with hepatocyte-specific deletion of *Tsc1*, a negative regulator of mTORC1. While the normal-chow diet-fed *Tsc1*−/− animals displayed evidence of hepatic and systemic insulin resistance, their livers did not show signs of steatosis, and the corresponding levels of hepatic triglyceride and expression of lipogenic genes (e.g., SREBP1, ACLY, FASN) were similar to those of the wild-type littermates. These findings suggest that constitutive mTORC1 activation per se is not sufficient for the development of steatosis. We further tested the effects of rapamycin in two independent models of steatosis to determine if mTORC1 activity is necessary for triglyceride accumulation in hepatocytes. Six weeks of high-fat (Surwit) diet in the wild-type mice gave rise to hypertriglyceridemia, hyperglycemia, hyperinsulinemia and steatosis that are commonly associated with the metabolic syndrome. *Pten* deletion in hepatocytes results in profound hepatomegaly and steatosis as previously reported [15] [16]. In both models, hepatic Akt2 has been shown to be the key mediator of lipid accumulation [40] [41]. Two weeks of rapamycin treatment significantly reduced mTORC1 activity but failed to suppress hepatic triglyceride levels in either model. Instead, there was a trend towards higher expression of lipogenic genes (e.g., SREBP1c) following rapamycin treatment. These observations led us to conclude that mTORC1 is neither necessary nor sufficient for steatosis.

mTORC1 is a key effector downstream of Akt involved in cell growth and proliferation [24]. Activation of either Akt or mTORC1 can lead to tumor formation [42] [43]. However, in the liver, these two kinases appear to have opposing effects on lipid accumulation. While the *Pten*-null livers developed profound steatosis, the *Tsc1*-null livers had low TG stores. This phenotypic difference correlated closely with their relative Akt and mTORC1 activities and suggested that the *Tsc1*−/− hepatocytes could be protected from steatosis due to the feedback suppression of Akt by mTORC1. In support of this, the *Tsc1*−/− livers were resistant to high-fat diet-induced steatosis, and treatment with rapamycin abolished this ‘protection’ resulting in hepatic TG accumulation that was equivalent to that seen in the wild-type hepatocytes under high-fat diet condition (Figure 8). Further, rapamycin led to the inhibition of mTORC1 and S6K1 resulting in the de-repression of Akt. Moreover, steatosis can be induced in the *Tsc1*−/− hepatocytes with the expression of Myr-Akt (Figure 7). These observations highlight the strong association between the balance of Akt and mTORC1 activities and the development of steatosis. When Akt dominates over mTORC1 (e.g., *Pten*−/−), steatosis ensues, whereas when mTORC1 overshadows Akt (e.g., *Tsc1*−/−), fat deposition is suppressed. Other models of Akt suppression in the liver (i.e., deletion of hepatic insulin receptor or Akt2) also result in a reduction in TG accumulation along with glucose intolerance similar to that of the *Tsc1*−/− mice [44] [41]. Thus, inhibition of hepatic Akt activity by any number of mechanisms leads to total hepatic insulin resistance. On the contrary, increasing Akt function in hepatocytes by direct (i.e., *Pten* ablation, expression of Myr-Akt, high-fat diet) or indirect (i.e., de-repression by rapamycin) means promotes lipogenesis and steatosis. These findings support our conclusion that the protective effect of mTORC1 from diet-induced steatosis is mediated via the inhibition of Akt signaling and underscore the potential for targeting Akt pharmacologically in the treatment of steatosis.

Rapamycin is commonly used as an immunosuppressant following renal transplant, and more recently, its analogs have gained FDA approval for use in human tumors such as renal cell carcinoma and subependymal giant cell astrocytoma. Reports of rapamycin-induced glucose intolerance and dyslipidemia are consistent with our observations. However, steatosis is not consistently associated with the use of rapamycin in humans. We reasoned that the degree of hepatic TG varies with the effects of rapamycin on Akt activity. Sarbassov et al. reported that Akt activity varies with the concentration and duration of rapamycin treatment such that acute rapamycin alleviates S6K1 feedback inhibition of Akt, but at higher concentrations and/or at longer exposure, rapamycin can inhibit Akt by reducing mTORC2 complex formation [45]. Thus, the net result of chronic rapamycin administration on Akt is difficult to predict. The rapamycin regimens that were used in our experiments effectively suppressed mTORC1 without significantly inhibiting Akt activity. Consequently, the hepatic TG contents remained either unchanged (Figure 4) or enhanced (Figure 8) correlating with the level of Akt signaling and the balance between Akt and mTORC1. When used for a protracted period (e.g., 2 mg/kg daily for 42 days), Chang et al. reported that diet-induced steatosis was suppressed in wild-type mice treated with rapamycin [34]. While Akt activity was not reported in the study, we speculate that their regimen may have inhibited Akt resulting in lowered TG accumulation. A more detailed examination of this relationship and the balance between Akt and mTORC1 activities in human NAFLD are potentially informative.

Insulin promotes lipid synthesis through the induction of SREBP1c and its target genes [46]. PI3K is the dominant signaling node responsible for insulin action, and a number of effectors downstream of PI3K have been implicated in hepatic lipid synthesis including Akt, PKC-ζ and PKC-λ [47]. While high-fat diet leads to obesity and hyperinsulinemia, in the liver, HFD induces a lipogenic response through the up-regulation of SREBP1c and down-regulation of ATGL that is accompanied by an increase in glucose kinase and a decrease in PEPCK (Figure 9). These changes are consistent with augmented fat synthesis and storage at the expense of utilizing glucose and suppressing gluconeogenesis during the state of over-nutrition. To the contrary, activation of mTORC1 leads to a metabolic switch from glucose utilization towards fat utilization in the liver similar to that seen during fasting or caloric restriction. Compared to wild-type littermates, hepatocytes with the loss of *Tsc1* have reduced SREBP1c and GK expression while ATGL and PEPCK were elevated, and these differences were recapitulated when fed a high-fat diet. Importantly, rapamycin had opposing effects on the expression of these metabolic enzymes suggesting that mTORC1 plays a critical role on the regulation of hepatic lipid and glucose metabolism. Based on the metabolic gene expression profile, the effects of rapamycin, when given at a non-Akt suppressing dose, resembles that of HFD feeding in promoting energy storage at the expense of burning glucose (e.g., markedly elevated glucose kinase and repressed PEPCK expression, Figure 9). Correspondingly, the liver responds to mTORC1 activation with a rapamycin-sensitive increase in PGC1α, a key regulator of mitochondrial biogenesis, which is normally induced under fasting conditions to facilitate glucose production. Thus, the *Tsc1*−/− model highlights the novel function of hepatic mTORC1 in enhancing gluconeogenesis while limiting the accumulation of triglyceride by promoting lipid utilization.

Although mTORC1 has been implicated in *de novo* lipogenesis in cells [10], the lack of TG accumulation in the *Tsc1*-null livers when challenged with HFD suggests that mTORC1 is not the primary ‘driver’ of steatosis *in vivo*. Instead, we surmise that mTORC1 serves to ‘fine-tune’ Akt signaling in the regulation of hepatic lipid metabolism. The mechanism of Akt-dependent steatosis involves a number of down-stream effectors including GSK3β and FoxO1. Akt phosphorylates GSK3β and FoxO1 to inhibit their activities, and in the *Tsc1*−/− livers, these proteins were hypo-phosphorylated (i.e., in the active state) (Figures 7, 9). GSK3β limits lipogenesis by phosphorylating mature SREBP1 and promoting its proteasomal degradation through binding with the Fbw7 ubiquitin ligase [48]. The effects of FoxO1 on hepatic SREBP1 are less clear with reports showing mixed results [49] [50] [51]. However, FoxO1 also regulates ATGL expression in promoting triacylglycerol hydrolysis [37], and ATGL was found to be significantly elevated in the *Tsc1*−/− livers (Figure 9). Loss-of-function mutations of ATGL have been associated with TG accumulation in patients with neutral lipid storage disease [52]. In summary, our data suggest that mTORC1 suppresses lipid accumulation through its feedback inhibition of Akt, which, in turn, modulates lipogenic and lipolytic activities through its effectors, GSK3β and FoxO1. These results also highlight the *in vi*vo relevance of the mTORC1-Akt feedback mechanism in regulating hepatic lipid metabolism and energy balance.
